# Comprehensive Transcriptome Analysis of Stem-Differentiating Xylem Upon Compression Stress in Cunninghamia Lanceolata

**DOI:** 10.3389/fgene.2022.843269

**Published:** 2022-03-03

**Authors:** Zekun Zhang, Huiyuan Wang, Ji Wu, Yandong Jin, Shengwu Xiao, Tao Li, Xuqinq Liu, Hangxiao Zhang, Zeyu Zhang, Jun Su, Jingzao Liu, Xiaoyan Wang, Yubang Gao, Xiangqing Ma, Lianfeng Gu

**Affiliations:** ^1^ College of Forestry, Fujian Agriculture and Forestry University, Fuzhou, China; ^2^ College of Forestry, Basic Forestry and Proteomics Research Center, Fujian Agriculture and Forestry University, Fuzhou, China; ^3^ Taining State-owned Forest Farm, Taining, China; ^4^ Sanming Forestry Bureau, Sanming, China

**Keywords:** compression wood, Chinese fir (Cunninghamia lanceolata (C.L.), PacBio Iso-seq, LncRNA - long noncoding RNA, lignin biosynthesis, cellulose biosynthesis

## Abstract

Compression wood (CW) in gymnosperm brings great difficulties to wood industry using wood as raw materials since CW presents special wood structure and have different physical and chemical properties from those of normal wood (NW). Chinese fir (*Cunninghamia lanceolata*) is widely distributed in China. However, global transcriptome profiling of coding and long non-coding RNA in response to compression stress has not been reported in the gymnosperm species. In this study, we revealed that CW in Chinese fir exhibited distinct morphology and cytology properties compared with those of NW, including high lignin content, thick and round tracheid cells. Furthermore, we combined both PacBio long-read SMRT sequencing (Iso-Seq) and Illumina short-read RNA-Seq to reveal the transcriptome in stem-differentiating xylem (SDX) under different time points (2, 26, and 74 h) upon compression stress in NW, CW, and OW (opposite wood), respectively. Iso-Seq was successfully assembled into 41,253 *de-novo* full-length transcriptome reference (average length 2,245 bp). Moreover, there were striking differences in expression upon compression stress, which were involved 13 and 7 key enzyme genes in the lignin and cellulose synthesis, respectively. Especially, we revealed 11 secondary growth-related transcription factors show differential expression under compression stress, which was further validated by qRT-PCR. Finally, the correlation between 6,533 differentially expressed coding genes and 372 differentially expressed long non-coding RNAs (lncRNAs) indicates that these lncRNAs may affect cell wall biogenesis and xyloglucan metabolism. In conclusion, our results provided comprehensive cytology properties and full-length transcriptome profiling of wood species upon compression stress. Especially we explored candidate genes, including both coding and long non-coding genes, and provided a theoretical basis for further research on the formation mechanism of CW in gymnosperm Chinese fir.

## Introduction

Wood plays a very important role in human society. The wooden logs can be processed into plates and sticks, which are used in the manufacture of various furniture and buildings (Ramage et al., 2017). At the same time, the high cellulose content of wood makes it an essential source of raw materials for papermaking and biofuels production (Ragauskas et al., 2006). In natural state, trees develop a special woody tissue due to gravity stimulation or mechanical stress such as strong wind, heavy snow, unstable soil, *etc*. (Du and Yamamoto, 2007). The cross-section of the trunk shows a kind of eccentricity, and one side of the concentric circle is wider (Ruelle, 2014). This special wood structure is called reaction wood (RW) (Dadswell and Wardrop, 1949), which can correct the growth of slanted branches and stems (Sinnott, 1952; Wilson and Archer, 1977).

The RW in angiosperms is called Tension Wood (TW) (Dadswell and Wardrop, 1949), which is formed in the area of the trunk under tension stress (the upper side of the inclined stem) (Scurfield, 1973). Although TW usually has vessels with smaller pore diameters, it has higher cellulose content and lower hemicellulose and lignin content (Dadswell and Wardrop, 1955). TW fiber forms an inner gelatinous cell wall layer (G-layer) which is composed of highly crystalline cellulose (Furuya et al., 1970). However, not all angiosperms have the G-layer (Okuyama et al., 1994; Clair et al., 2006; Sultana et al., 2010). Plant cell walls are composed of the middle lamella, primary cell wall, and secondary cell wall (Dey and Brinson, 1984). The secondary cell wall is usually divided into 3 layers due to different microfibril angle (MFA) (Bidlack, 1992). The S2 layer of TW cell wall has a very low MFA (Donaldson, 2008).

In model species poplars, researchers have carried out a lot of studies on the RW formation through transcriptome sequencing (Yeh et al., 2007; Wang et al., 2013; Martin et al., 2014; Chen et al., 2015a; Chen et al., 2015b; Kerwin, 2021; Ren et al., 2021; Yu et al., 2021). C2H2 transcription factor (TF) (PtaZFP2) negatively regulates the molecular response of poplar and is critical in the process of plants adapting to mechanical stimuli (Martin et al., 2014). In addition to C2H2, 2 TFs (HSFB3-1 and MYB092) in the stems of *Populus tomentosa* were significantly induced in TW and have five common directly targeted genes (Liu et al., 2021). A significantly induced TF (PtrLBD39) from curved poplar trees was revealed from stem xylem based on transcriptome sequencing. Transcriptomic analysis of CRISPR-based *PtrLBD39/22* double mutant revealed that PtrLBD39 indirectly regulates 10 cell wall component genes in TW responsive (Yu et al., 2021). Potential regulatory role during the forming of RW in mature xylem tissues of *Populus tomentosa* is revealed by constructing transcriptome-level regulatory relationship between lncRNAs, miRNAs, and mRNAs in NW, TW, and OW (opposite wood), respectively (Qiu et al., 2015).

The RW in gymnosperms is formed on the underside of the inclined stem (Scurfield, 1973). This special wood structure is called Compression Wood (CW) (Dadswell and Wardrop, 1949). The cell wall of CW tracheid cells is thicker and round in shape (Yoshizawa and Idei, 1987). The content of lignin in CW is increased, and the MFA in the S2 layer is higher (Westing, 1965; Timell, 1982; Donaldson and Singh, 2013). Chinese fir (*Cunninghamia lanceolata*) is an important artificial timber forest species in China, and its area and stock volume account for the highest proportion of artificial forests (Duan et al., 2016; Chen et al., 2020). Thus, Chinese fir forests occupy an extremely important position in the sustainable development of China’s forestry.

With the development of sequencing technology, the sRNA library of Chinese fir from leaves, stems, seeds, seedlings, and calli was sequenced using Illumina Next Generation Sequencing (NGS) to identify a broad set of sRNAs, including miRNAs, rasiRNAs, and tasiRNAs (Wan et al., 2012). Small RNA sequencing of vascular cambium reveals the expression changes of miRNA156 and miRNA172 from dormancy to active growth (Qiu et al., 2015). In addition to miRNA, mRNA degradation played a key post-transcriptional regulation during the release of primary dormancy of Chinese fir seed (Cao et al., 2016). Currently, the research of Chinese fir non-coding RNA mainly focuses on the small RNA, and the research on the long non-coding RNA has not been carried out yet.

RW brings great difficulties to the standardized production of the wood industry due to unevenly distributed density of wood material (Ormarssonand et al., 2000; Wimmer and Johansson, 2014). The goal of these industries is to obtain high-purity cellulose (Santos et al., 2013). However, the high lignin content of the CW in gymnosperms has brought great trouble to remove it in pulp processing and biofuel production (Obst, 1990; Himmel et al., 2007). Thus, the studies on the regulation of CW formation at the molecular level are critical in Chinese fir. The process of Chinese fir producing CW provides an excellent model for us to study the secondary growth of Chinese fir (Pilate et al., 2004; Foston et al., 2011). However, the studies of Chinese fir based on sequencing technology have not been widely carried out due to the lack of genomic reference sequence so far. In this study, we combined both long-read SMRT sequencing (Iso-Seq) and short-read RNA-Seq to reveal the transcriptome in stem-differentiating xylem (SDX) of Chinese fir under different time points (2, 26, and 74 h) upon early compression stress in NW, CW, and OW, respectively. Firstly, we revealed the cytology characteristics changes in response to compression stress in Chinese fir. Especially, we revealed transcriptome profile for lignin, cellulose biosynthesis-related genes, and TFs of SDX in response to compression stress. Finally, we found that lncRNAs have potential regulatory functions in this process. Both the transcript profiles and the regulatory network of lncRNA in this study provided many candidate genes for further revealing the formation of CW in Chinese fir. Our research not only provides a foundation for further research on the molecular mechanisms for formation of CW, but also provides new clues to improve wood quality in angiosperms by genetic manipulation.

## Materials and Methods

### Plant Material

**FIGURE 1:**
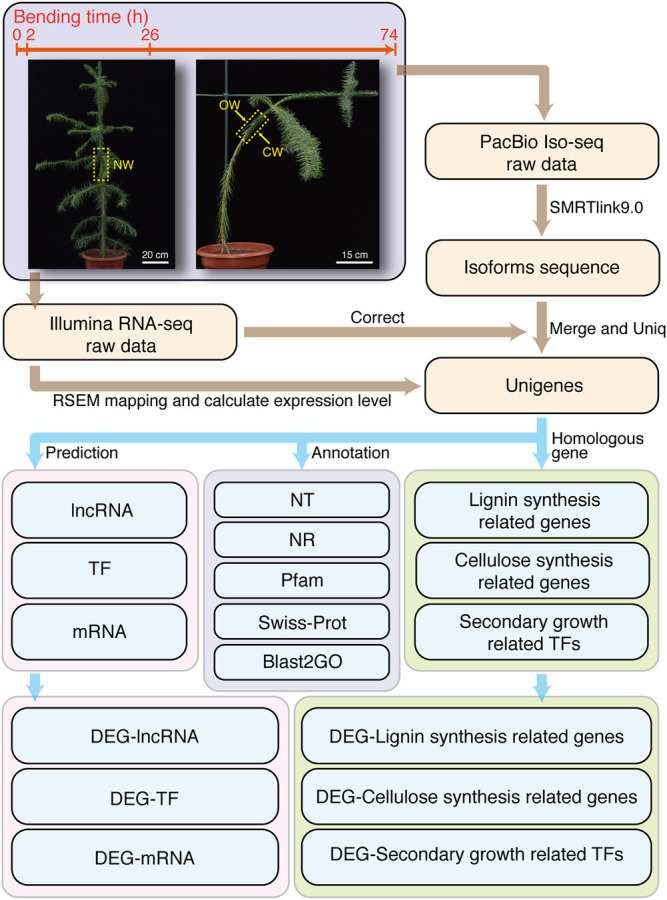
Flow chart of experimental design under artificial bending and Bioinformatic workflow. The bending treatment time point and the material collection time point are 0, 2, 26, and 74 h, respectively.

Chinese fir was grown in the greenhouse of Fujian Agriculture and Forestry University. The growth condition was maintained at ∼25°C with 16 h light/8 h dark photoperiod. We selected Chinese fir with relatively uniform height (1.2–1.3 m) as experimental material. The NW group without bending treatment was used as control (Figure 1). We also performed artificial bending treatment following previous method (Paux et al., 2005) to induce the generation of CW in Chinese fir. The angle between the 0.5–0.7 m height of the trunk and the vertical direction is 45° (Figure 1). At 10:00 AM on 21 October 2020, SDX tissue samples without bending treatment were collected as NW. The bending treatment of all groups started at 8:00 AM on 22 October 2020. The collection time for 2, 26, and 74 h treatment groups is 10:00 AM on October 22, 23, and 25, respectively (Figure 1).

After the bending treatment, the SDX samples were collected immediately. About 20 cm of the trunk at the bent part of the plant was cut. Then we peeled off the bark and scraped the SDX on the upper (the side far from the ground, marked as OW) and lower sides (the side near the ground, marked as CW) of the bent stem. The materials of NW were collected at the equivalent position. All the samples were immersed in liquid nitrogen, and stored at −80°C until RNA isolation.

### Anatomy Observation of *Cunninghamia lanceolata*

We collected wood stems from Chinese fir plants that grow straight and lean, respectively. The bark was peeled off, and then the stems were cut into 0.8–1 cm. One-half number of the small fresh stems were cut into 100-micron slices using Leica rotary microtome. The 100-micron slices were dehydrated using alcohol and tert-butanol. Then the water-removed slices were used for scanning electron microscope observation. At the same time, the other small stems were dehydrated and transparentized using alcohol and tert-butanol, and then embedded using paraffin wax. The tissue wax block was cut into 12-micron paraffin slices using Leica rotary microtome. The paraffin slices were dewaxed by xylene and stained using safranin O and fast green. Then the slices were used for upright microscope observation.

### Ribonucleic Acid Extraction and Quality Evaluation

The SDX tissue materials were grinded and crushed using the motorized tissue grinders. We extracted the total RNA by using the RNAprep Pure Plant Kit (Polysaccharides & Polyphenolics-rich) (Tiangen, Beijing, China) according to manual. Residual DNA was removed using RNase-free dnase I (Tiangen, Beijing, China). The quantity and purity of RNA samples were evaluated using the NanoDrop 2000 spectrophotometer to meet the requirements of library construction (1.8 < OD260/280 < 2.2). The RNA integrity and precise concentration were detected (using Agilent 2,100 RNA 6000 Nano kit) in Agilent 2,100 Bioanalyzer System. RNA samples with RNA Integrity Number (RIN) ≥ 7.5, RNA concentration ≥300 ng/μL, and total RNA amount ≥2 μg were used in the downstream experiment. In total, 14 RNAs from 7 experimental groups (NW, 2hCW, 2hOW, 26hCW, 26hOW, 74hCW, and 74hOW) with two biological repeats were extracted for RNA sequencing and qPCR validation.

### Construction of PacBio Iso-Seq Libraries and Sequencing

In total, 14 PacBio Iso-Seq libraries were constructed using the SMARTer PCR cDNA Synthesis Kit (Clontech, #634925). Total RNA samples were reverse-transcribed into first-strand cDNA. The distribution characteristics of amplified products were confirmed by electrophoresis and Agilent 2,100. The cycle number of PCR amplification of cDNA was determined by the pre-amplification to obtain a sufficient amount of cDNA. The purified PCR amplicons were used to construct the SMRT sequencing library using the Template Prep Kit (PacBio, #100–259–100). The sequencing library was purified with PB beads and quantified using Qubit, for which quality control was performed using Agilent 2,100. In brief, 14 libraries with 1–10 kb insert sizes were constructed and sequenced by PacBio Sequel II.

### Construction of Illumina Libraries and Sequencing

In this study, 14 libraries were also constructed for second-generation transcriptome sequencing. SDX mRNA was enriched by Oligo (dT)25 magnetic beads. In first-strand synthesis reaction buffer, with elevated temperature, the rRNA-depleted RNA fragments are cut into smaller fragments using divalent cations. By using random primer and reverse transcriptase, the first-strand cDNA was synthesized. By using buffer, dNTPs (containing dUTP instead of dTTP), RNaseH, and DNA polymeraseⅠ, the double-strand cDNA was synthesized. Then, double-strand cDNA was purified. Next, the purified double-stranded cDNA was performed terminal repair, poly-A tail addition, sequencing adapter connection, and the fragment size selection. The sequencing library was obtained by PCR enrichment. The sequencing library was initially quantified using Qubit, and the effective concentration was accurately quantified by qPCR. In total, 14 strand-specific cDNA libraries were constructed for Paired-End RNA Sequencing with reads length of 150 nt.

### Full-Length Reference Transcriptome

We used SMRT Link 9.0 command-line tool isoseq3 program (version 3.3.0) to process subreads (https://github.com/PacificBiosciences/IsoSeq). First, we used the ccs module (using the polish parameter) of the isoseq3 program to generate the circular consensus sequencing (CCS read). Next, we used the lima module of the isoseq3 program to remove the primers of the CCS sequence. We used the refine module to trim poly-A tails and remove concatemer sequences. After this step, we obtained full-length non-concatemer reads (FLNC reads). Finally, we used the cluster module to cluster the FLNC sequences and obtain the redundant isoforms sequence. After the combination of the FASTA files from above 14 processed Iso-Seq libraries, CD-HIT-EST software (Huang et al., 2010) was used to remove redundant sequences using following parameter: -c 0.99 -M 0 -T 0 -G 0 -aL 0.90 -AL 100 -aS 0.99 -AS 30. To improve the quality of isoforms sequences (removed redundant), we used Illumina reads to correct isoforms sequences by LoRDEC software (Salmela and Rivals, 2014) using following parameter: -t 5 -b 200 -e 0.4 -T 40 -k 21 -s 3. Finally, we again used CD-HIT-EST software (-c 0.95 -M 0 -T 0) to cluster isoforms sequences after error correction for redundancy removal. In brief, we obtained non-redundant, non-chimeric, full-length unigenes, which makes subsequent analyses more accurate. The flowchart for the bioinformatics analysis is shown in Figure 1.

### Functional Annotation for Unigenes

For exhaustive annotation of above full-length unigenes, we used BLAST software (version 2.5.0) (Altschul et al., 1997) to search against NR (NCBI Non-redundant Protein Sequence) (Wheeler et al., 2007), NT (Nucleotide Sequence database), and Swiss-Prot (Apweiler et al., 2004), respectively. The NT, NR, and Swiss-Prot annotations correspond to unigenes using blastn, blastx, and blastp, with E-value <1*10^–6^. Moreover, the unigenes were also annotated based on the Pfam (protein family) database (Finn et al., 2014) using HMMSCAN module of HMMER software (version 3.3.2) (Potter et al., 2018). Finally, Gene Ontology (GO) (Ashburner et al., 2000) terms were assigned to unigenes by Blast2GO software (Conesa and Gotz, 2008).

### Identification of Long Non-Coding Ribonucleic Acid and Transcription Factor

ORF of unigenes with a base number >200 nt was predicted using getorf module from EMBOSS software (version6.3.1) (Rice et al., 2000). The unigenes whose ORF amino acids were less than 120 were extracted. We used CNCI (Sun et al., 2013), CPC2 (Kang et al., 2017), and PLEK (Li et al., 2014) to identify lncRNA. The sequences predicted by the three software were intersected to obtain high confidence lncRNAs.

For TF identification, we removed lncRNAs from unigenes firstly. Then the CDS region of unigenes was identified and translated into a protein sequence using ESTScan 3.0, which was provided by the Plant Transcription Factor database (PlantTFDB 5.0) from PlantRegMap (Tian et al., 2020). The TF families were identified by mapping the protein sequence to PlantTFDB 5.0.

### Quantification of Unigene Upon Bending Treatment

Clean data from 14 Illumina RNA-seq libraries were mapped to full-length unigenes using the RSEM software (--bowtie2, version 1.3.0) (Li and Dewey, 2011). We obtained genes read count using rsem-calculate-expression module of RSEM software from the mapping results. The Spearman’s correlation coefficient (SCC) between replicates in each experimental group was calculated as a measure of repeatability. Then, the differentially expressed unigenes among different treatment groups were calculated using DESeq2 (version 1.26.0) (Love et al., 2014) using *p*-value < 0.05 and fold change>=2.0 as cutoff. According to the classification of lncRNA and TF, the differentially expressed lncRNAs and differentially expressed TFs were obtained.

### Co-Expression Analysis of Differentially Expressed Long Non-Coding Ribonucleic Acid and Coding Gene

We further classified differentially expressed unigenes into lncRNAs and coding genes. Pearson correlation coefficient (PCC) between lncRNA and coding gene was calculated and selected to construct the gene co-expression network by Cytoscape software (version 3.8.0) (Kohl et al., 2011) using PCC >0.9 as cutoff.

### Gene Ontology Enrichment Analysis

Using the clusterProfiler package (version 4.0.2) (Wu et al., 2021) with *p*-value < 0.05 as cutoff, we performed GO functions enrichment analysis on differentially expressed coding genes from the 6 treatment groups and from the co-expression network in 20 GO terms, respectively.

### Quantitative Real Time-Polymerase Chain Reaction Validation and Expression Analysis

We synthetized the first-strand cDNA using Hifair Ⅲ SuperMix plus, and 1 μg of total RNA was used. The cDNA was used as template, Hieff qPCR SYBR Green Master Mix was used for quantitative reverse transcription-polymerase chain reaction on Mx3005P QPCR system (Stratagene, Santa Clara, CA). Primers for the selected 12 genes (genes related to the lignin and cellulose synthesis pathway, TFs related to the regulation of secondary growth, and predicted lncRNAs) were designed using NCBI Primer-BLAST tool. The primers are listed in Supplementary Table S1. We chose the EF1α (elongation factor1-alpha) gene as an internal reference for normalization. This gene is stably expressed in Chinese fir across different tissues and organs. The final 20 μL reaction contained: primers with final concentration of 0.2 μM, 2 μL template cDNA(1:20 dilution), and 1X Power SYBR Green dye. PCR amplification conditions: 95°C for 5 min, 40 cycles of 95°C for 10 s, 60°C for 30 s. After amplification, a thermal denaturation cycle was performed to determine the dissociation curve for verification of amplification specificity: 95°C for 15 s, 60°C for 15 s, and 95°C for 15 s. All qRT-PCR reactions were repeated 4 times.

## Results

### Cytological Observation of Normal Wood, Compression Wood, and Opposite Wood in Chinese Fir

**FIGURE 2:**
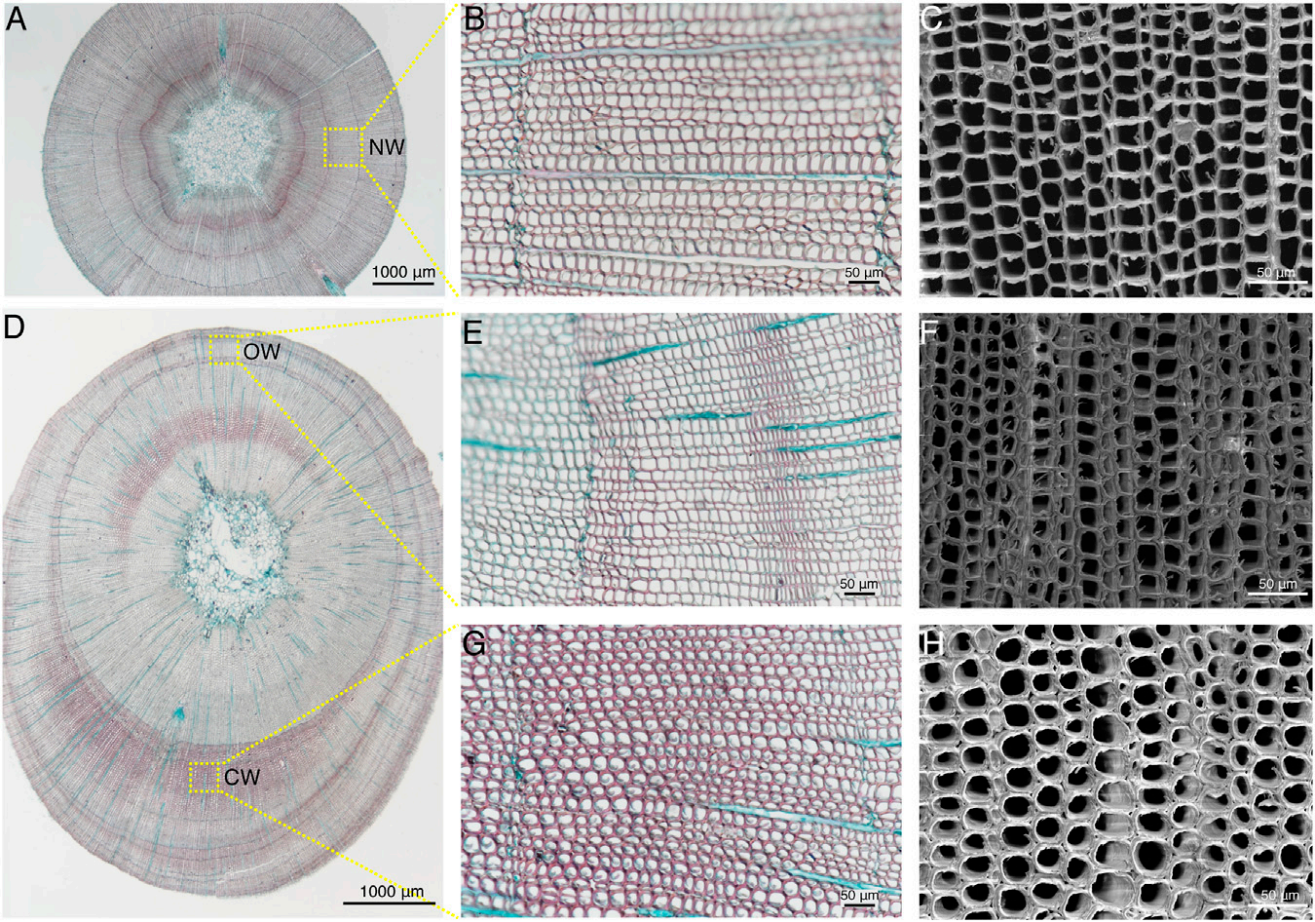
Cytological structure of compression wood (CW) formation in *C. lanceolata*. **(A,D)** Cross sections of straight stem and leaning stem, which were double-stained with Safranin O and Fast Green FCF, respectively. Scale bars = 1,000 μm. **(B,E,G)** Sections of NW, OW, and CW double-stained with Safranin O and Fast Green FCF, respectively. Scale bars = 50 μm. **(C,F,H)** Scanning electron microscopy images of the xylem cross-sections of NW, OW, and CW, respectively. Scale bars = 50 μm.

In order to observe cell structure of the NW, OW, and CW in xylem of Chinese fir, the paraffin sections from straight and leaning trunks were observed by upright microscope, and the fresh slices after dehydrate were observed by scanning electron microscopy (Figure 2). The width of the entire xylem of the NW is relatively uniform (Figure 2A). The tracheids in the NW were quadrilateral or polygonal and arranged tightly according to the paraffin section under the X200 field of view (Figure 2B), which was further confirmed under X500 scanning electron microscope (Figure 2C). Compared with the straight Chinese fir, the leaning Chinese fir showed obvious eccentric growth, and the xylem of the CW was wider than that of the OW (Figure 2D). Safranin and fast green staining revealed that the color of CW is redder than OW. The lignin content of the xylem in CW is higher than that of OW, which was consistent with the results of earlier studies in Pinus and Chinese fir (Nanayakkara et al., 2009; Li et al., 2021). The tracheid morphology in the OW was consistent with the tracheid morphology in NW (Figure 2E), which was further confirmed under the X500 scanning electron microscope (Figure 2F). The tracheids in the CW were obviously elliptical or round, with intercellular spaces, and the tracheid cell wall also showed a certain degree of thickening (Figure 2G), which were consistent with the results of earlier studies in European yew, red cedar, and Norway spruce (Burgert et al., 2004). The special tracheid morphology in the CW was further confirmed under X500 scanning electron microscope (Figure 2H).

### Construction of Full-Length Transcriptome Using PacBio Iso-Seq

The full-length transcriptome libraries of 14 SDX samples from Chinese fir were sequenced using PacBio Sequel II platform. A total of 382.78 G subreads bases were generated, which included a total of 236,935,007 reads of insert (ROI). The statistics of PacBio raw data are shown in Supplementary Table S2. The average number of ROI generated by each library was 16,923,929 and the average read quality was >99.9%. After using the SMRTlink9.0 command-line tool to process subreads from the ZMW, we obtained a total of 4,136,486 circular consensus sequencing (CCS) reads, and the average number of CCS reads generated by each library was 295,463. The CCS sequence was processed with 5′, 3′ sequencing primer removal and chimeric sequence removal. Finally, a total of 3,682,735 FLNC sequences were obtained. The average FLNC reads number in each library was 263,052. The FLNC reads were clustered by using the cluster module of isoseq3 software resulted in a total of 415,423 high-quality isoform sequences.

### Generation of Non-Redundant Full-Length Unigene

We merged 415,423 high-quality isoform sequences from 14 PacBio full-length transcriptome libraries to perform the first de-redundancy for removing redundant sequences with sequence similarity ≥99% using CD-HIT-EST software. In total, there are 139,318 high-quality sequences remaining. Subsequently, the filtered high-quality sequences were further corrected using LoRDEC software and the short-read sequence generated by Illumina RNA-Seq. The corrected sequence was again removed redundant sequences by using the CD-HIT-EST software with a similarity ≥95%. In total, 41,253 high-quality sequences with an average length of 2,245 bp were obtained as full-length unigenes for consequent analysis.

### Integrative Annotation of 41,253 Full-Length Unigenes

The 41,253 unigenes were searched for NT, NR, and Swiss-Prot databases using BLAST for functional annotation. There were 25,453, 34,823, and 27,207 genes which obtained functional annotations from NT, NR, and Swiss-Prot, respectively. Moreover, 32,134 and 26,767 unigenes were annotated using HMMSCAN and BLAST2GO, respectively. The statistics of the full-length unigenes annotations were listed in Supplementary Table S3. GO classifications of the full-length unigenes were shown in Supplementary Table S4.

### Identification of Long Non-Coding Ribonucleic Acid and Transcription Factor

In total, there were 41,225 unigenes remaining after removing the unigenes sequences whose base number was less than 200. Subsequently, the ORF of the remaining sequences was predicted using EMBOSS-getorf to exclude the sequences whose ORF is greater than 120. There were 5,527 remaining unigenes for lncRNA identification. In total, there were 4,949, 5,363, and 4,049 lncRNAs based on the result from CNCI, CPC2, and PLEK, respectively. Finally, a total of 3,602 unigenes were annotated as lncRNAs using the common intersection from three software (Supplementary Table S5).

After removing 3,602 lncRNAs from 41,253 unigenes, we annotated TF using 37,651 unigenes according to PlantTFDB database. A total of 1,185 unigenes were annotated as TFs. The most abundant group of TF family was the bHLH family(93), followed by ERF(75), bZIP(74), Trihelix(74), C2H2(73), and C3H(73) family, respectively (Supplementary Table S6).

### Profiling Differentially Expressed Coding Genes

NGS technology has a greater sequencing depth than third-generation sequencing. Thus, we used the Illumina RNA-seq libraries to quantitatively analyze the expression of the full-length unigenes. The 14 SDX strand-specific mRNA transcriptome libraries were sequenced and generated a total of 324,215,605 paired-end reads. On average, each library produced 23,158,258 reads of 150 nt. The statistics of Illumina RNA-Seq data are shown in Supplementary Table S7. The SCC showed good repeatability and reliability between replicates in each experimental group (Supplementary Figure S1).

**FIGURE 3:**
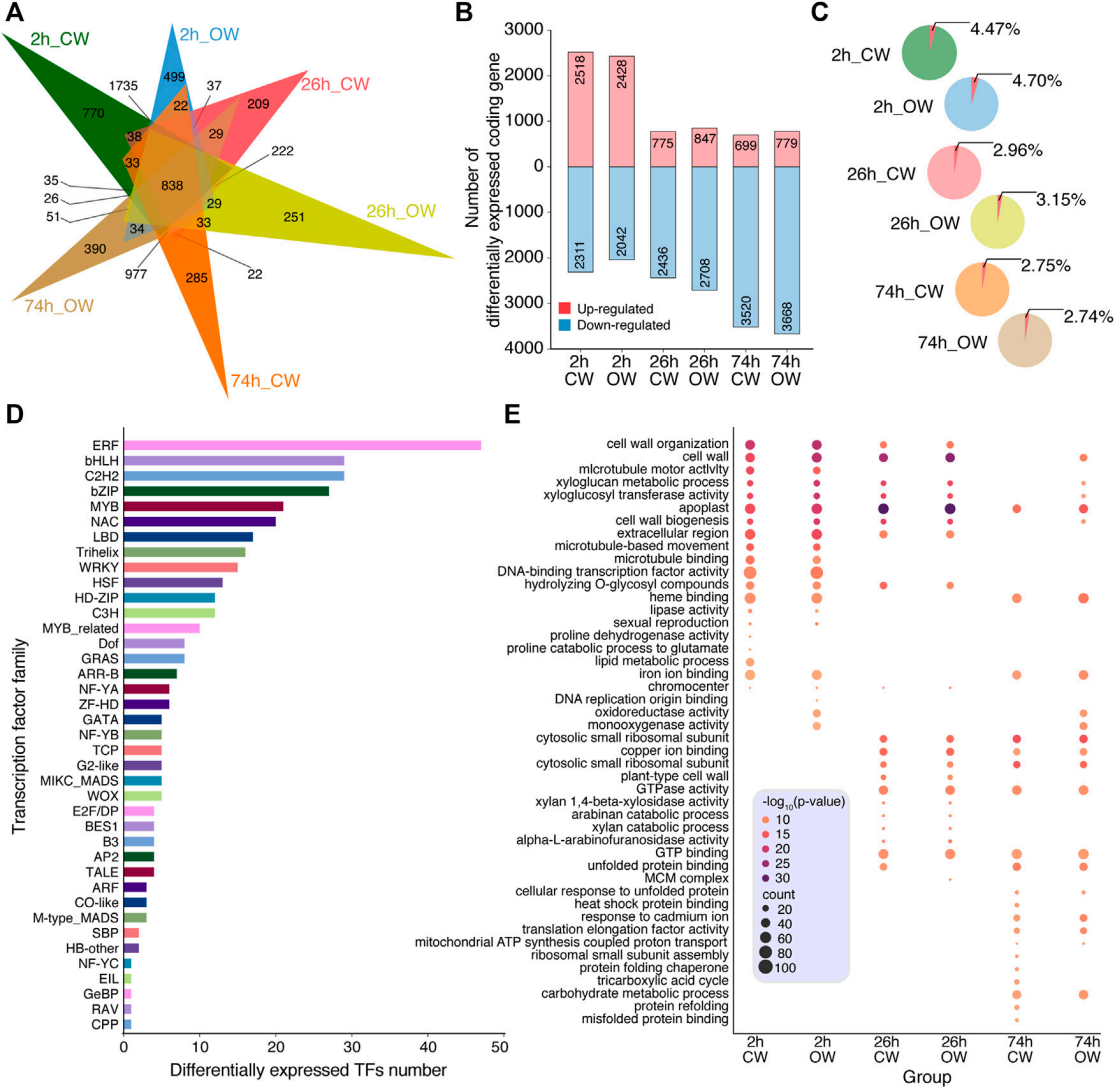
Profiling of differentially expressed coding genes. **(A)** Venn diagram of the DECGs among 6 treatment groups. **(B)** Histogram displays the up-regulated and down-regulated genes, which are marked with red and blue, respectively. **(C)** Pie chart presents the percentage of TFs in DECGs. **(D)** Histogram displays the classification of DETFs. **(E)** Bubble chart displays the GO enrichment analysis of DECGs.

According to the alignment of Illumina RNA-Seq, we obtained all the expression levels of unigenes in 14 samples using RSEM software (Supplementary Table S8). The differentially expressed unigenes between the control group (NW) and the six treatment group (2 h_CW, 2 h_OW, 26 h_CW, 26 h_OW, 74 h_CW, 74 h_OW) were identified using DESeq2 R package (*p*-value < 0.05, log_2_FoldChange> 1). From six pairwise comparisons, we obtained the differentially expressed coding genes (DECGs), which included 838 DECGs in all comparisons (Figure 3A). Compared with the NW group, the number of the DECGs detected in the 2 h_CW, 2 h_OW, 26 h_CW, 26 h_OW, 74 h_CW, and 74 h_OW was 4,829, 4,470, 3,211, 3,555, 4,219, 4,447, respectively (Supplementary Table S9). Overall, the number of DECGs in the 2 h treatment group was the largest, followed by the 74 h treatment group, and the least number of DECGs was in the 26 h treatment group. The number of DECGs unique to the six treatment groups was 770, 499, 209, 251, 285, 390, respectively (Figure 3A). The number of unique DECGs in the 2 h treatment group was the largest, and the up-regulated DECGs accounted for about 53% of the total DECGs (Figure 3B). The number of up-regulated DECGs in the 26 h treatment group and 74 h treatment group was significantly reduced, accounting for 24 and 17% of the total DECGs, respectively. For down-regulated DECGs, the number of down-regulated DECGs in the 26 and 74 h treatment groups was higher than that in the 2 h treatment group, and the 74 h treatment group had the largest number of down-regulated DECGs (Figure 3B). In addition, the number of DECGs obtained by CW compared with OW gradually increased in a time-dependend manner upon compression stress (Supplementary Figure S2, Supplementary Table S10).

Among the DECGs, we found a total of 371 differentially expressed TFs (DETFs) (Supplementary Table S11). The percentage of DETFs in DECGs among the six treatment groups was 4.47, 4.70, 2.96, 3.15, 2.75, and 2.74%, respectively (Figure 3C). The proportion of DETFs in CW and OW samples gradually decreases as time increases upon bending treatment (Figure 3C). The 2 h treatment group had the highest proportion of DETFs. Among the DETFs, the number of ERF, bHLH, C2H2, bZIP, MYB, and NAC families was 47, 29, 29, 27, 21, and 20, respectively (Figure 3D).

GO enrichment analysis was performed on the DECGs (*p*-value < 0.05). The top 20 GO terms with the most significant *p*-value were retained (Supplementary Table S12). The DECGs of 2 h_CW and 2 h_OW were enriched in GO terms, including cell wall organization, cell wall, xyloglucan metabolic process, xyloglucosyl transferase activity, cell wall biogenesis, *etc*. (Figure 3E). In addition to the above terms, the 26 h_CW and 26 h_OW treatment groups were enriched in plant-type cell wall, xylan 1,4-β-xylosidase activity, arabinose catabolic process, xylan catabolic process, *etc*. (Figure 3E). For the 74 h treatment group, only the 74 h_OW treatment group had DECGs enriched in cell wall, xyloglucan metabolic process, cell wall biogenesis, xyloglucosyl transferase activity, *etc*. (Figure 3E). Xyloglucan is a component of plant hemicellulose (Fry, 1989). Arabinose is often combined with other monosaccharides, and exists in the form of heteropolysaccharides in hemicellulose, pectic acid, the heartwood of coniferous trees, and in some glycosides (Maki-Arvela et al., 2011). According to the GO enrichment analysis of the DECGs in the six treatment groups, we revealed that the DECGs related to the SDX of Chinese fir are involved in the process of plant cell wall production and plant hemicellulose catabolism after the artificial bending treatment. This might be one of the reasons that Chinese fir produces reaction wood tissue to resist bending stress and restore straight growth.

### Differential Expression of Genes Related to Cell Wall Synthesis and Transcriptional Regulation

**FIGURE 4:**
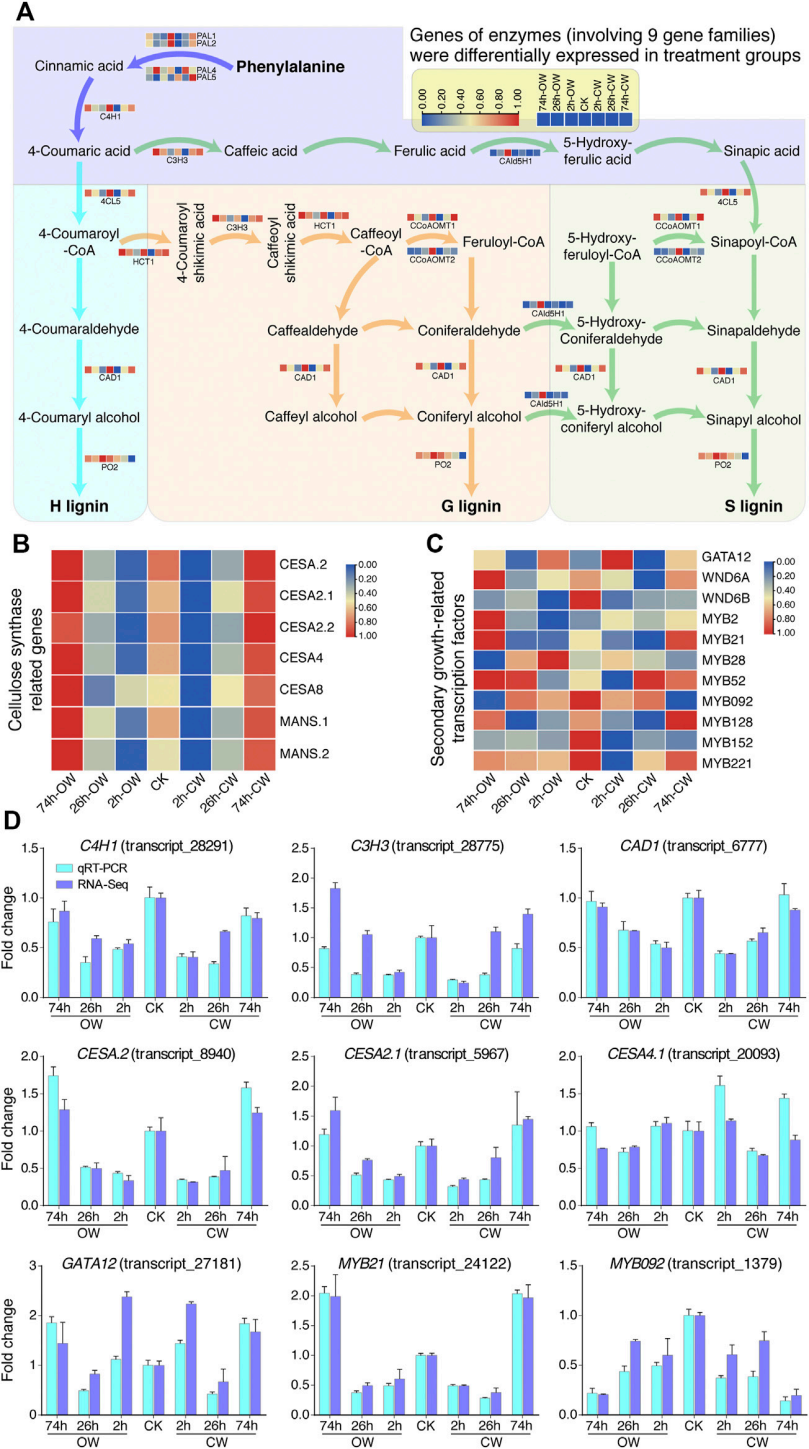
Expression patterns of DEGs related to cell wall synthesis. **(A)** Differential gene expression in lignin synthesis pathway. **(B)** Differential gene expression in cellulose synthesis pathway. **(C)** Differential TF expression in plant secondary growth. In **(A,B)**, and **(C)**, the gene expression was varied from high (red) to low (blue) value. **(D)** Histogram displays qRT-PCR verification of genes involved in cell wall synthesis and transcriptional regulation.

Chinese fir generates CW in response to stress, and the lignin and cellulose content of its xylem have changed. We analyzed the expression of the genes involved in the lignin and cellulose synthesis pathways and the TFs related to the plant secondary growth based on homologous genes from *Populus trichocarpa*. According to RNA-Seq result, we revealed the differential expression levels of lignin, cellulose-related genes, and secondary growth-related TFs (Figure 4).

Previous studies have found that the phenylalanine biosynthesizes three kinds of lignin-subunits (H- subunit, G-subunit, S-subunit) through a metabolic grid consisting of 11 enzyme families and 24 metabolites (Chen et al., 2015a; Wang et al., 2018; Liu et al., 2021). In gymnosperms, the G lignin monomer accounts for most of its lignin content. In this study, we found that genes of 13 enzymes (involving 9 gene families) were differentially expressed in treatment groups (Figure 4A, Supplementary Table S13). Differentially expressed genes involved in lignin synthesis included four phenylalanine ammonia-lyases genes (*PAL1*, *PAL2*, *PAL4*, *PAL5*), one cinnamic acid 4-hydroxylase gene (*C4H1*), one 4-coumaric acid 3-hydroxylase gene (*C3H3*), one 4-coumaric acid: CoA ligase gene (*4CL5*), one cinnamyl alcohol dehydrogenase gene (*CAD1*), one hydroxycinnamoyl-CoA shikimate hydroxycinnamoyl transferase gene (*HCT1*), two caffeoyl-CoA O -methyltransferases genes (*CCoAOMT1*, *CCoAOMT2*), one coniferaldehyde 5-hydroxylase gene (*CAld5H1*), and one peroxidase gene (*PO2*), respectively. Most of the genes involved in the lignin monomer synthesis pathway were generally down-regulated first, and then restored to normal expression levels or up-regulated (Figure 4A).

For genes related to the cellulose synthesis pathway (Chen et al., 2015a; Liu et al., 2021), we found 5 cellulose synthase genes (*CESA.2*, *CESA2.1*, *CESA2.2*, *CESA4*, and *CESA8*) and two glycosyltransferase family mannan synthase genes (*MANS.1* and *MANS.2*) are differentially expressed upon bending treatment (Figure 4B, Supplementary Table S14). Except for the down-regulation of *CESA8* in 26 h_OW, the other genes showed an obvious down-regulation at 2 h stage first, and then recovered to a certain extent at 26 h groups. Finally, their expression levels were up-regulated at 74 h groups.

Two key TFs PtrHSFB3-1 and PtrMYB092 regulate cell wall biosynthesis during the formation of TW in poplar (Liu et al., 2021). PtrMYB092 can directly activate 11 monolignol genes. In the study of the poplar wood formation process, PtrGATA12, as a nuclear localization transcription activator, coordinately regulates the biosynthetic pathway of secondary cell wall components (Ren et al., 2021). In our study, we also detected 11 TFs genes (Figure 4C, Supplementary Table S15), including *GATA12*, *WND6A*, *WND6B*, *MYB2*, *MYB21*, *MYB092*, *etc*., which are differentially expressed at different treatment groups. The qRT-PCR analysis of these genes confirmed the expression changes detected by RNA-Seq (Figure 4D). The expression analysis of genes related to secondary growth provides valuable clues for explicating the molecular mechanism of CW formation.

### Correlation Between Differentially Expressed Long Non-Coding Ribonucleic Acids and Differentially Expressed Coding Genes

**FIGURE 5:**
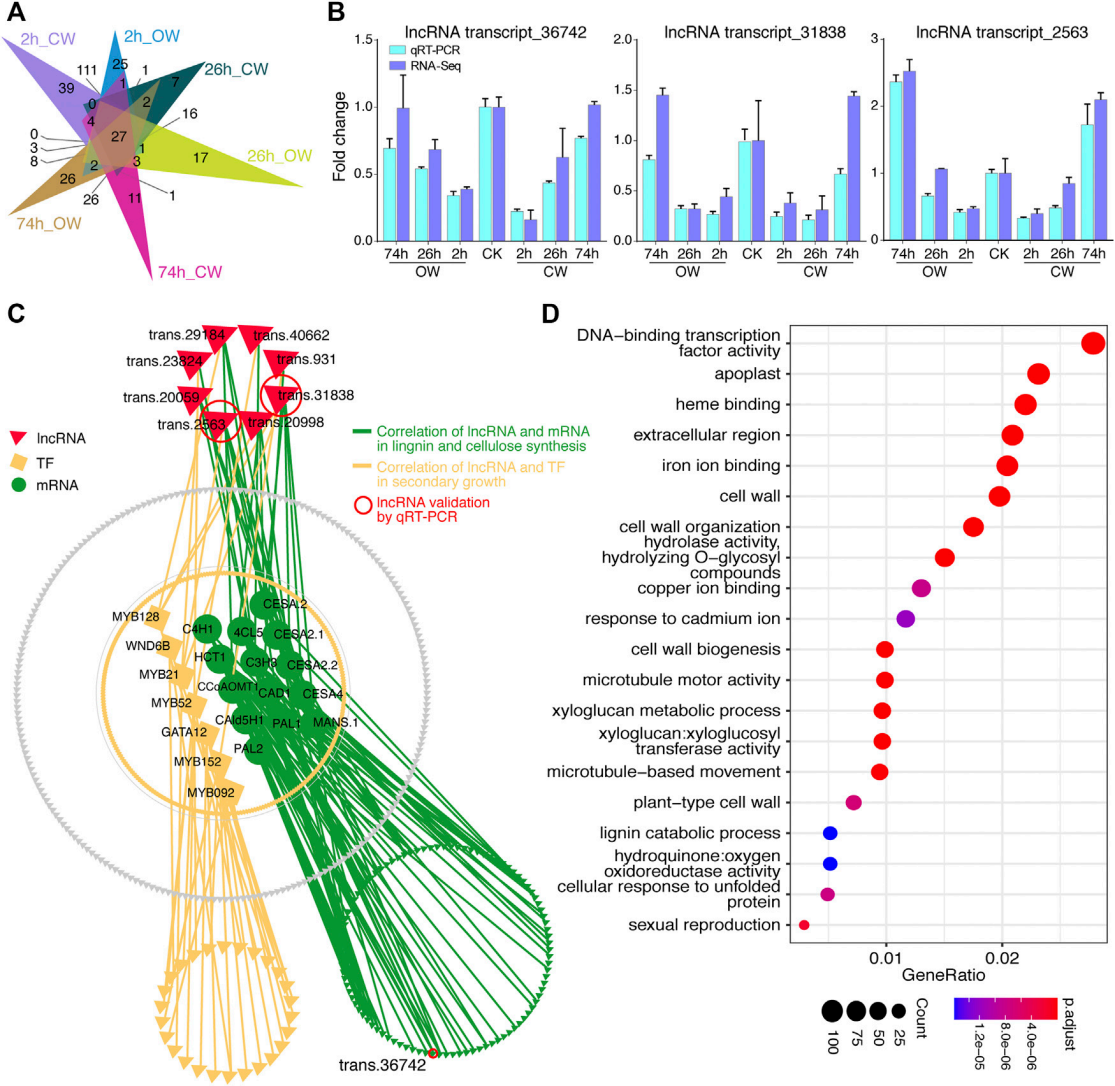
Differential expression analysis of lncRNA. **(A)** Venn diagram shows DElncRNAs of 6 treatment groups. **(B)** Histogram displays qRT-PCR verification of lncRNAs. **(C)** Correlation network diagram between DElncRNAs and DECGs. **(D)** Bubble chart presented GO enrichment analysis of DECGs, which were correlated with lncRNA.

Among 9,785 differentially expressed unigenes during the artificial bending process, of which 434 are predicted lncRNAs, and the remaining 9,351 are DECGs (including 371 TFs). The differentially expressed lncRNAs (DElncRNAs) in CW and OW samples at 2, 26, and 74 h treatment groups were shown in Figure 5A (Supplementary Table S16). The expression changes of these lncRNAs were confirmed by qRT-PCR (Figure 5B). And the results of qRT-PCR showed that the expression trends of 3 lncRNAs were consistent with those obtained from RNA-Seq, indicating the accuracy of the RNA-Seq data.

According to the DECGs and DElncRNAs expression, we calculated the PCC (Supplementary Table S17), and the correlation between the expression of DElncRNAs and DECGs was shown through the network diagram (Figure 5C). We revealed a significant correlation (r > 0.9) between 372 DElncRNAs and 6,533 DECGs (6,263 mRNA, 270 TF). GO enrichment analysis was performed on these 6,533 coding genes (*p*-value < 0.05, keep the 20 GO terms with the most significant *p*-value, Figure 5D, Supplementary Table S18). Coding genes presenting correlation with lncRNA were enriched in GO terms such as cell wall, xyloglucan metabolism, cell wall organization, cell wall biogenesis, xyloglucosyl transferase activity, plant-type cell wall, *etc*. (Figure 5D). This may imply that lncRNA is involved in regulating the process of compression stress response in Chinese fir.

## Discussion

In recent years, many studies have used RW as a model to discover the key genes in the process of wood formation. For example, 9,684 differentially expressed genes were found among TW, OW, and NW transcriptome in *Betula platyphylla* (Wang et al., 2014). In *Populus tomentosa*, 2,417 genes were differentially transcribed between TW and NW (Chen et al., 2015a). In *Betula luminifera*, global transcriptome profiling analysis revealed 13,273 differentially regulated unigenes during the early stage of TW formation (Cai et al., 2018). In addition, 384 up-regulated and 410 down-regulated genes were identified in *Catalpa bungei*, which provides a new basis for explaining the formation of TW (Xiao et al., 2020b). However, most of the studies were carried out in angiosperm, and only a few research were conducted on gymnosperms. Especially the formation of CW in Chinese fir remains unknown.

For non-model organisms, the emergence of transcriptome sequencing technology makes it possible to reveal the special gene expression characteristics without reference genome information (Collins et al., 2008; Ekblom and Galindo, 2011). However, subsequent analysis based on second-generation sequencing technology will encounter great difficulties, especially in the process of assembling the transcripts (Grabherr et al., 2011; Pertea et al., 2015). Moreover, different transcript isoforms often show high sequence similarity (Martin and Wang, 2011). In this study, we used PacBio Iso-Seq technology to obtain full-length transcriptome of SDX from Chinese fir under artificial bending as reference unigene set. A total of 415,423 full-length sequences from 14 PacBio Ise-Seq libraries were filtered and retained to 41,253, which was further corrected by the short reads from the same batch of RNAs. This makes the full-length unigenes unprecedentedly complete and accurate, and represents the transcriptome characteristics of the Chinese fir under compression stress. The precise and complete unigenes also enable us to obtain more accurate annotation. The high precision full-length unigenes can provide great advantage for the research of Chinese fir.

In our study, we obtained superior transcription maps of Chinese fir NW, CW, and OW SDX under different bending times of 2, 26, and 74 h, respectively. The average length of unigenes we obtained is greater than the 497 bp obtained by previous study (Qiu et al., 2013). In this study, a total of 9,351 DECGs were identified during the process of artificial bending, which indicates that the gene expression of the SDX has changed considerably during this process of CW formation in Chinese fir. In addition, with the continuation of the bending time, the number of DECGs decreased and then increased, which is consistent with previous study in wild-type poplars (Pomies et al., 2017). In another study, the number of differentially expressed genes (DEGs) gradually increased in the 6 h, 48 h, and 7 d groups in *Betula luminifera* (Cai et al., 2018). Therefore, we speculate that the number of DEGs is highest in the early stage (0.5 h, 2 h) under artificial bending, and with the continuation of the bending time, it showed a decreasing trend and then increased.

Moreover, the up-regulated genes in angiosperms accounted for more or nearly half of the DEGs in the initial stage of artificial bending, such as 0.5, 2, or 6 h (Pomies et al., 2017; Cai et al., 2018). While at 24, 48, 72 h, 7 d, 90 d, the DEGs accounted for a larger proportion of down-regulated genes (Chen et al., 2015a; Pomies et al., 2017; Cai et al., 2018; Xiao et al., 2020a; Xiao et al., 2020b). Our study showed the same trend, in the 2 h group, up-regulated genes accounted for 53%, while in the 26 h, 74 h groups, down-regulated genes accounted for a larger proportion (Figure 2B). This indicates that the up-regulated genes are dominant in the early phase, while down-regulated genes become increasingly important in the late phase.

In addition, we found that as the bending time increases, the proportion of TF in the DECGs gradually decreases, and the ERF TF family accounts for the largest number of DETFs, which are consistent with previous study in wild-type poplars (Pomies et al., 2017). In addition, study on the secondary growth of poplar has proved that the TF PtrGATA12 directly or indirectly regulates some TFs (WND6A, WND6B, MYB152, and MYB21) to affect the formation of secondary cell walls (Ren et al., 2021). In this study, we explored the continuous expression of TFs related to secondary growth. The *GATA12* expression showed a trend of U-shaped curve (Figure 3D). Study on the formation of poplar TW has shown that TF PtrMYB092 can transactivate 11 monoglycol synthesis pathway genes (Liu et al., 2021). In our study, a downward trend was observed in the expression of *MYB092* with the bending time increases, which might affect the expression of monoglycol synthesis pathway genes.

Previous studies have found extensive expression changes in lignin and cellulose synthesis pathway genes during the formation of RW (Paux et al., 2005; Wang et al., 2014; Chen et al., 2015a; Cai et al., 2018; Xiao et al., 2020a; Liu et al., 2021). In *Pinus pinaster*, the cellulose synthase-like A (*CSLA*) gene and the lignin biosynthesis genes, such as *PAL*, *C3H*, *4CL*, *CAD*, *HCT*, *CCoAOMT,* were up-regulated in CW (Villalobos et al., 2012). In *Buxus microphylla* var. *japonica*, the *PAL* gene is up-regulated in RW (Hiraide et al., 2016). In this study, we observed complex changes in lignin synthesis pathway genes, including *PAL4*, *PAL5*, *CAld5H1*, *CCoAOMT2*, and *PO2*, respectively. We speculated that the five genes with special expression trends in the lignin synthesis pathway might be critical in the formation of CW. Other DECGs involved in the synthesis of lignin and cellulose showed a similar trend of decreasing first and then increasing, which seems to be a prevalent response under artificial bending in Chinese fir.

In addition to coding RNA, non-coding RNA also plays an important role in plant transcriptional regulation (Liu et al., 2015; Liaquat et al., 2021; Zhang et al., 2021). Previous study has shown that transcriptome sequencing on the mature xylem tissues of NW, TW, and OW reveals 1,377 possible lncRNAs, of which 776 are differentially expressed in different tissue samples of poplar (Chen et al., 2015b). They report 389 differentially expressed lncRNAs that may target 1,151 genes through *trans*-regulation (Chen et al., 2015b). In our research, we found that there is a significant correlation between the expression levels of 372 DElncRNAs and 6,533 DECGs (r > 0.9) (Figure 5C). The lncRNA (transcript_31,838) and lncRNA (transcript_29,184) were significantly correlated with *CESA.2*, *CESA2.2*, *CESA4*, *MANS.1*, *MYB21*, *MYB128*, *PAL1*, and *CCoAOMT1*, respectively. Two lncRNAs may target these genes through *trans*-regulation in response to compression stress in Chinese fir. However, further transformation experiment should be carried out to validate the potential regulation in the future.

At present, we only investigated the expression level since Chinese fir genome was unavaiable. Once the genomic is available, our third-generation full-length transcriptome from 14 libraries can be used to investigate the post-transcriptional regulatory mechanism, including alternative splicing (AS) and alternative polyadenylation (APA) (Wang et al., 2017; Zhang et al., 2018; Yin et al., 2019) during the formation of Chinese fir CW in response to compression stress. This will further improve our understanding of the CW formation in Chinese fir. In addition, we expect the knowledge about the target sites of TFs and lncRNAs to obtain a more accurate regulatory network of Chinese fir under artificial bending by using ChIP-seq or DAP-seq when the genome of Chinese fir becomes available.

## Conclusion

In summary, we provide a full-length reference transcriptome of Chinese fir. Combined with Illumina NGS RNA-Seq, we obtained transcriptomes of the NW, CW, and OW SDX from Chinese fir (*Cunninghamia lanceolata*) under different bent times (2, 26, 74 h). By using 382.78 G subreads bases, we obtained 41,253 high-quality, non-redundant full-length unigenes with average length of 2,245 bp. Moreover, we revealed 20 full-length unigenes related to lignin and cellulose synthesis pathways, and 11 TFs related to secondary growth, which were differentially expressed at different treatment groups, indicating that these genes play a critical role in the process of producing CW in Chinese fir. At the same time, 3,602 possible lncRNAs were predicted, of which 434 lncRNAs were differentially expressed in different treatment groups. 372 DElncRNAs have a significant correlation with 6,533 DECGs. In conclusion, our research provides the first full-length SDX transcription data as an isoform-level reference for Chinese fir. All these findings not only provide a theoretical basis for exploring the regulation mechanism of the production of CW in Chinese fir, but also offer candidate functional genes resources for the production of CW.

## Data Availability

The names of the accession number(s) can be found below: PRJNA777739 and PRJNA777106. The datasets presented in this study can be found in the article/Supplementary Material.
