# Supplementary figures and images for: Comprehensive Transcriptome Analysis of Stem-Differentiating Xylem Upon Compression Stress in Cunninghamia Lanceolata
Source: Front Genet. 2022 Mar 3;13:843269. doi: 10.3389/fgene.2022.843269 (PMC8927042; doi:10.3389/fgene.2022.843269)

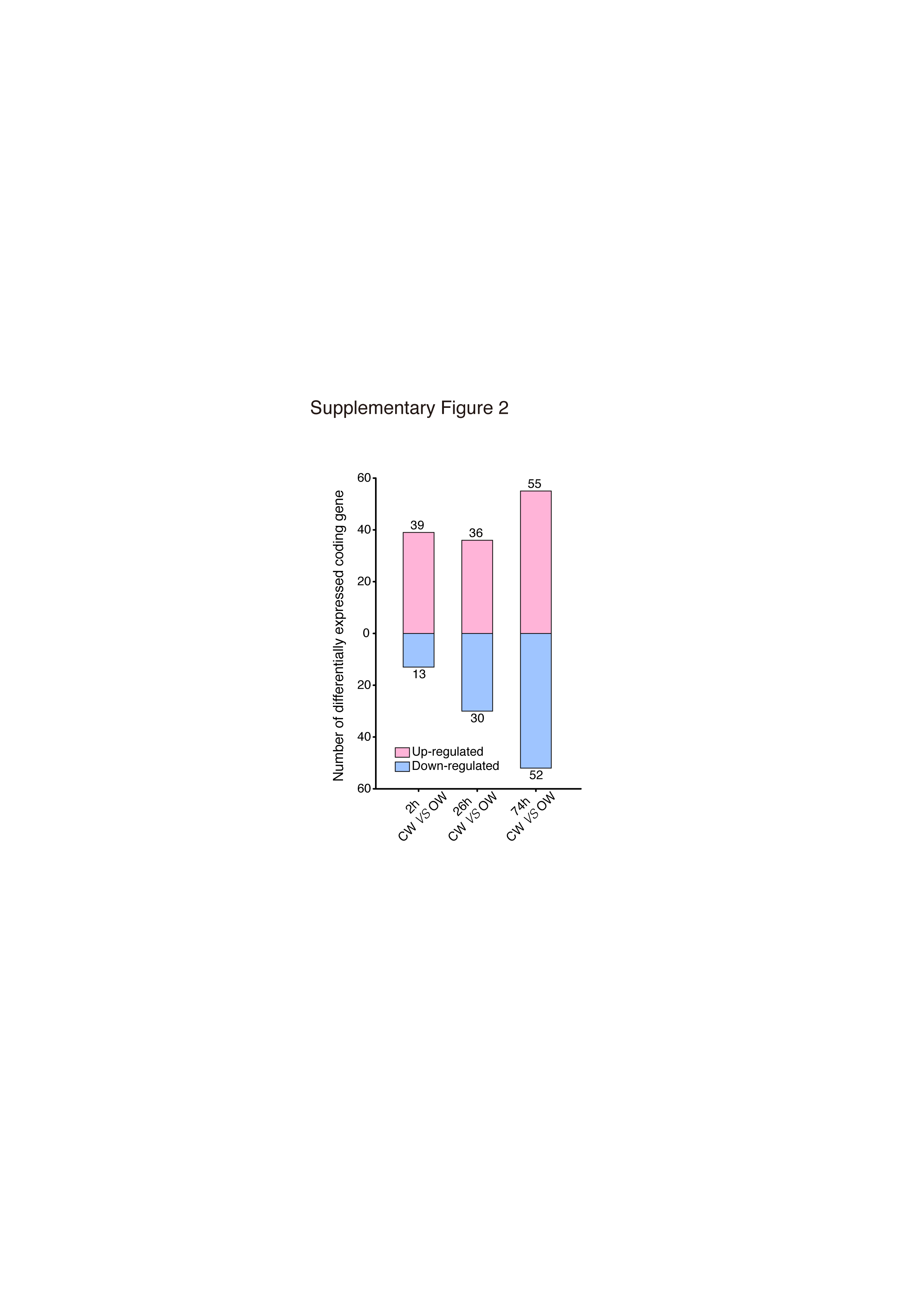

Supplement: Supplementary file 2 [file Image2.TIF]

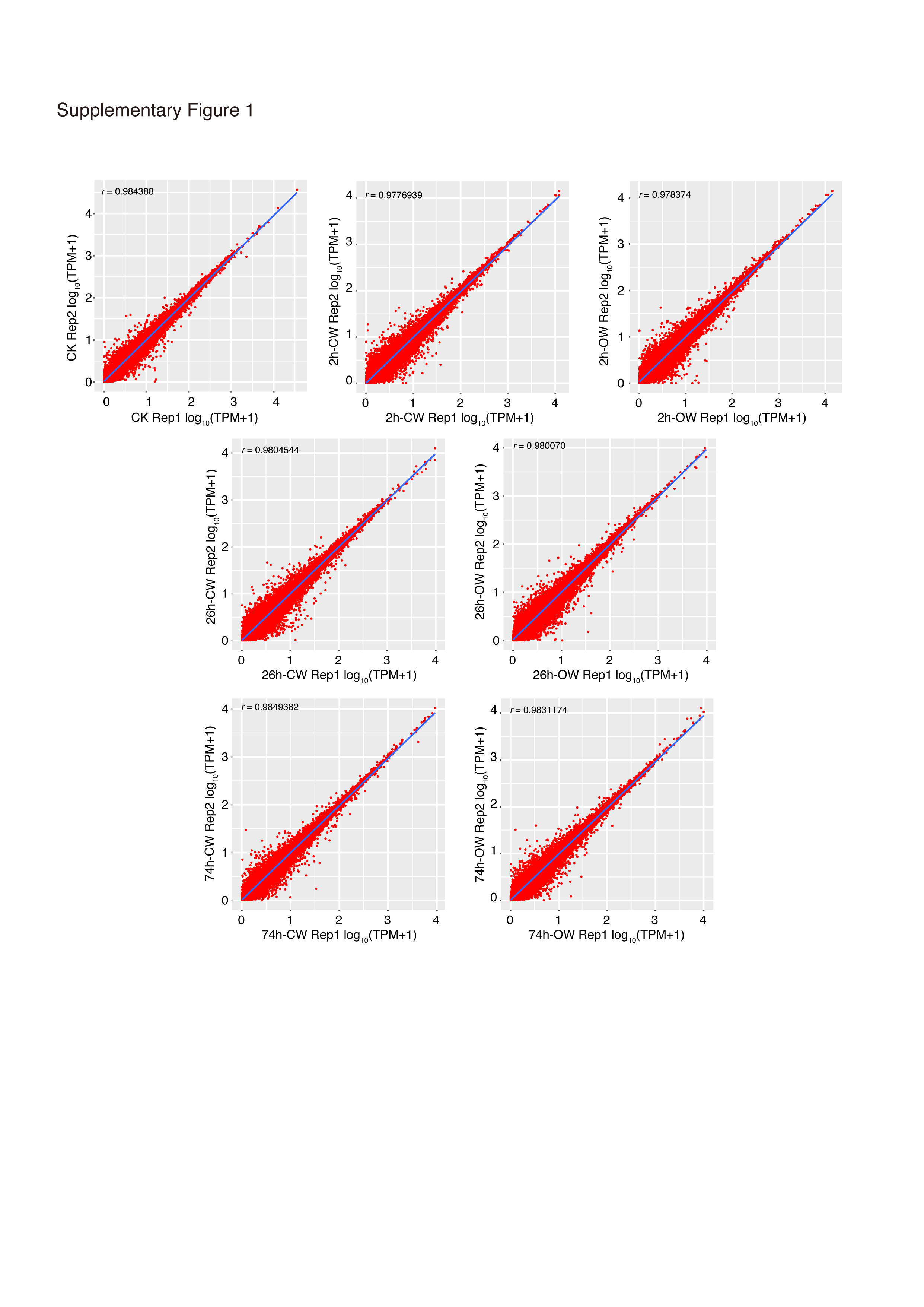

Supplement: Supplementary file 3 [file Image1.TIF]
